# Periappendiceal fluid collection on preoperative computed tomography can be an indication for interval appendectomy: a retrospective study

**DOI:** 10.1186/s13017-022-00437-9

**Published:** 2022-05-31

**Authors:** Shintaro Kanaka, Satoshi Mizutani, Yasuyuki Yokoyama, Takeshi Matsutani, Naoto Chihara, Akira Katsuno, Hideyuki Takata, Ryosuke Nakata, Keisuke Mishima, Yudai Wada, Takao Shimizu, Ryo Yamagiwa, Takahiro Haruna, Yuka Nakamura, Akira Hamaguchi, Nobuhiko Taniai, Hiroshi Yoshida

**Affiliations:** 1 Department of Gastrointestinal and Hepato-Biliary-Pancreatic Surgery, Nippon Medical School Musashikosugi Hospital, 1-383 Kosugimachi, Nakahara-ku, Kawasaki, Kanagawa 211-8533 Japan; 2 Department of Gastrointestinal and Hepato-Biliary-Pancreatic Surgery, Nippon Medical School, 1-1-5 Sendagi, Bunkyo-ku, Tokyo, 113-8602 Japan

**Keywords:** Acute appendicitis, Periappendiceal fluid collection, Management, Interval appendectomy

## Abstract

**Background:**

The treatment strategies for acute appendicitis, such as emergency appendectomy (EA), interval appendectomy (IA), and repeating nonoperative management (NOM), are controversial. In this study, we examined the preoperative factors that can be used to distinguish which patients should undergo IA.

**Methods:**

We retrospectively identified 902 patients who underwent surgery for appendicitis in our hospital from January 2010 to December 2021. Of these patients, 776 were included in this study. The patients were divided into two groups: those with a periappendiceal fluid collection (PAFC) on preoperative computed tomography (PAFC-positive group, *n* = 170) and those without a PAFC (PAFC-negative group, *n* = 606). In each group, we compared patients who underwent EA and IA.

**Results:**

In the PAFC-positive group, patients who underwent EA had a significantly higher postoperative complication rate than those who underwent IA (40.5% vs. 24.0%, *p* = 0.037). In the multivariate analysis, only the presence of PAFC was significantly associated with an increased risk of postoperative complications (odds ratio, 7.11; 95% confidence interval, 2.73–18.60; *p* < 0.001). The presence of PAFC alone was not significantly associated with an increased risk of IA or NOM failure (odds ratio, 1.48; 95% confidence interval, 0.19–11.7; *p* = 0.71). The rate of neoplasia on pathologic examination was significantly higher in the PAFC-positive than PAFC-negative group (7.6% vs. 1.5%, *p* < 0.001); the rate of carcinoma was also higher in the PAFC-positive group (2.4% vs. 0.17%, *p* = 0.02).

**Conclusions:**

The presence of PAFC on preoperative computed tomography was found to be a risk factor for postoperative complications but not IA or NOM failure. It was also correlated with neoplasia as the etiology of appendicitis. Therefore, PAFC positivity is useful as an indication for IA.

## Background

The diagnosis and treatment strategies for acute appendicitis are controversial. Clinically, acute appendicitis is classified as either complicated appendicitis or uncomplicated appendicitis [1]. However, it is often difficult to preoperatively determine the most appropriate management for acute appendicitis, such as emergency appendectomy (EA), interval appendectomy (IA), or repeating nonoperative management (NOM) [2]. In the present study, we considered the treatment strategy for acute appendicitis from the following three perspectives: prediction of which patients should avoid emergency surgery, prevention of failure of IA or NOM, and a malignant tumor as the etiology of the appendicitis. It is important to perform EA and IA in appropriate cases. We retrospectively examined preoperative computed tomography (CT) images and investigated whether the presence of a periappendiceal fluid collection (PAFC) on preoperative CT can be an indication for IA.

## Methods

**Fig. 1:**
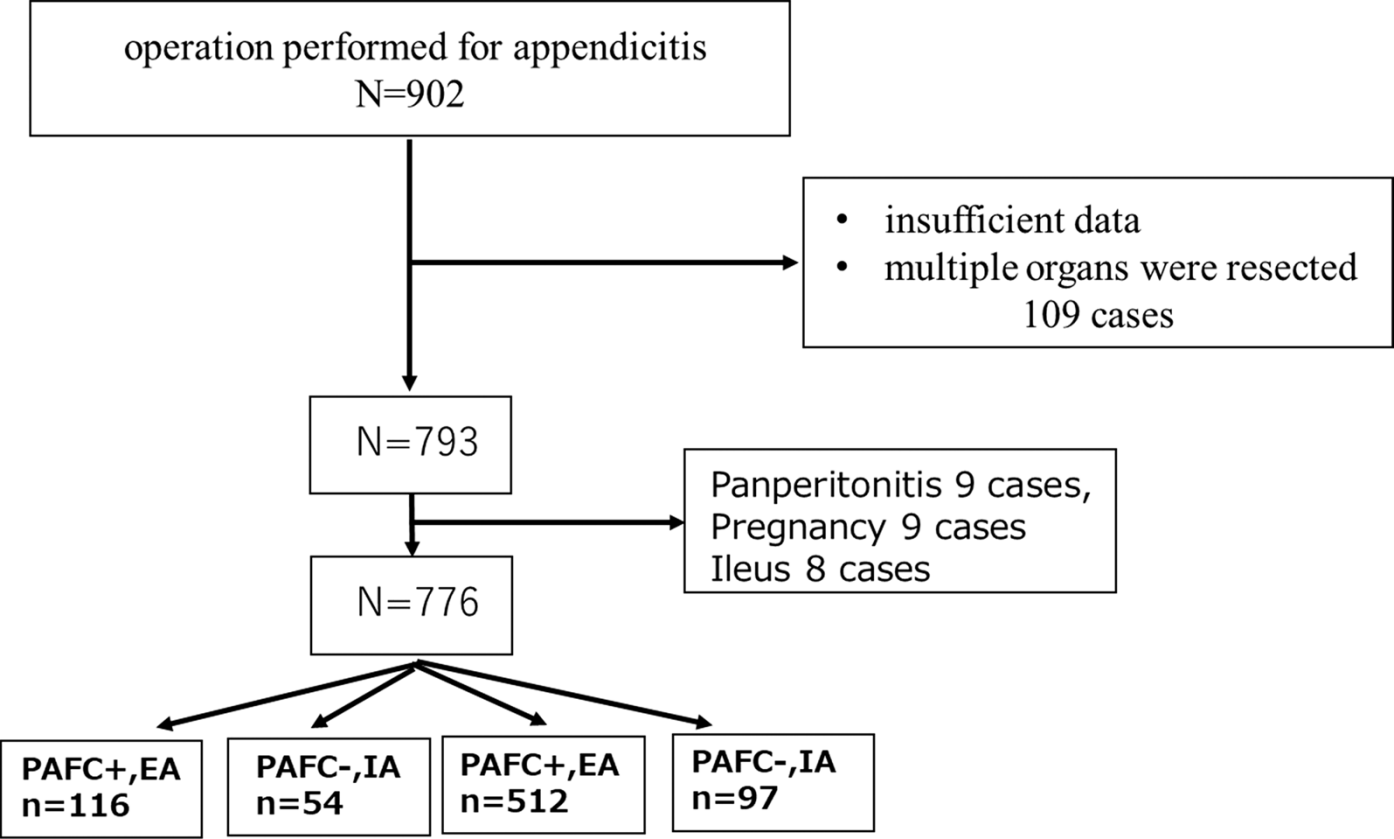
Flowchart of patient selection. *PAFC* periappendiceal fluid collection, *EA* emergency appendectomy, *IA* interval appendectomy

**Fig. 2:**
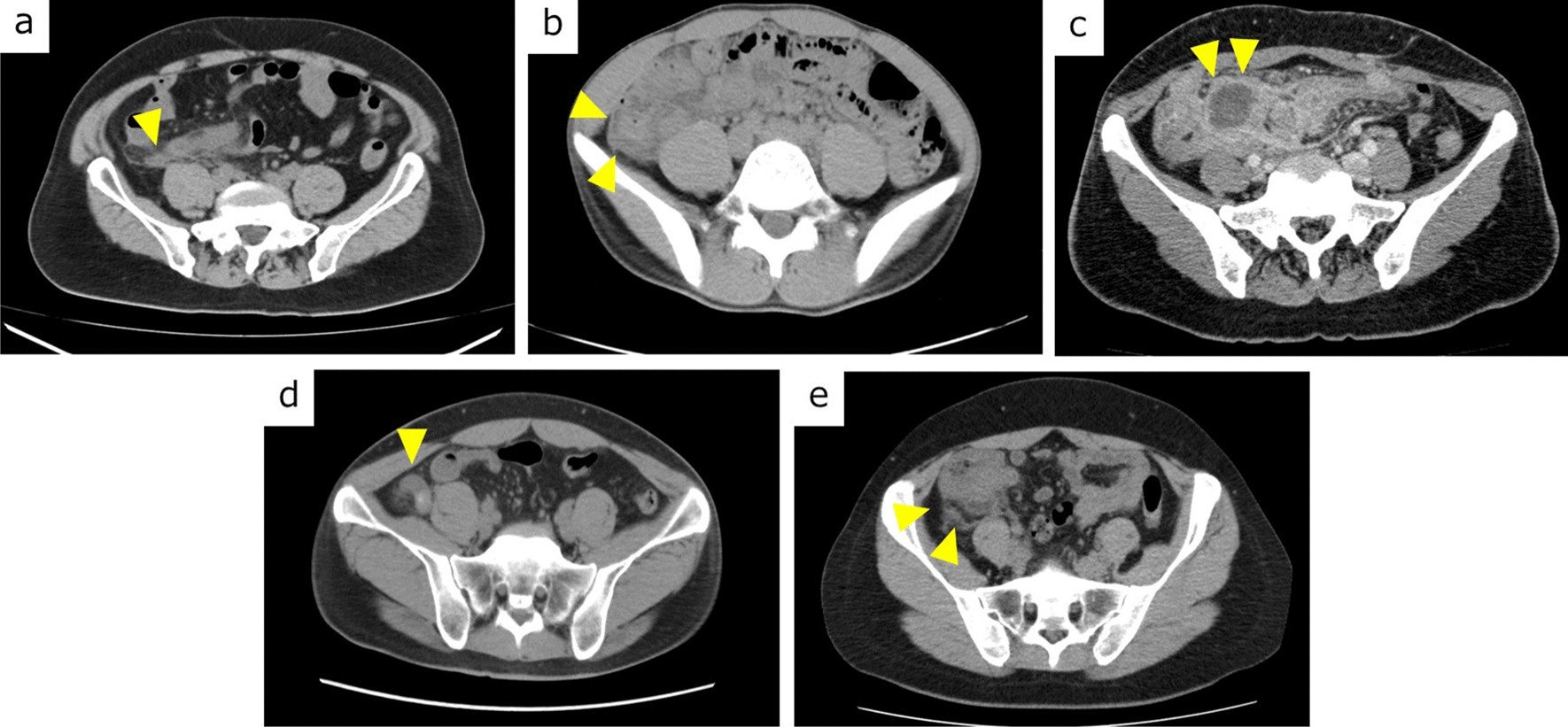
Computed tomography images. **a**, **b** Localized fluid collection around the appendix and cecum (arrowheads). **c** Localized abscess formation (arrowheads). **d**, **e** Swelling of the appendix and fat stranding are observed; however, no fluid collection is observed (arrowheads)

We retrospectively identified 902 patients who underwent surgery for appendicitis in our hospital from January 2010 to December 2021. Of these, 776 patients were included in this study. Patients with insufficient data and who underwent resection of multiple organs were excluded. Patients with conditions requiring emergency surgery, such as panperitonitis, ileus on preoperative CT, and pregnancy, were also excluded (Fig. 1). PAFC positivity was defined as obvious abscess formation and localized fluid retention around the appendix on preoperative CT (Fig. 2a–c), and PAFC negativity was defined as inflammation around the appendix but no fluid retention (Fig. 2d, e). Patients with nonlocalized fluid were considered to have panperitonitis and were thus excluded from the study. The patients were divided into two groups: the PAFC-positive group (*n* = 170) and the PAFC-negative group (*n* = 606). In each group, we compared patients who had undergone EA and IA. Cases of NOM failure were treated as complications of IA. The patients’ background and perioperative variables were collected from the medical charts. Postoperative complications were defined as Clavien–Dindo grade ≥ II complications within 30 days after surgery [3].

All statistical analyses were performed with EZR [4], which is a modified version of R Commander designed to add statistical functions frequently used in biostatistics. The *χ*^2^ test was performed to compare frequencies between groups. Differences in continuous variables between groups were compared using Student’s t test. If values did not show a normal distribution, the Mann–Whitney *U*-test was used. Two-sided *p* values of < 0.05 were considered statistically significant.

## Results

The PAFC-positive EA group comprised 116 patients, the PAFC-positive IA group comprised 54 patients, the PAFC-negative EA group comprised 509 patients, and the PAFC-negative IA group comprised 97 patients. Their characteristics are presented in Table 1. There was no significant difference in age, sex, body mass index, or operation time. In both the PAFC-positive and PAFC-negative groups, the rate of laparoscopic appendectomy was significantly higher in the patients who underwent IA than in those who underwent EA (PAFC-positive: 94.2% vs. 66.7%, *p* < 0.001; PAFC-negative: 94.8% vs. 84.3%, *p* = 0.01). Regarding postoperative complications, patients who underwent EA showed a significantly higher morbidity rate than patients who underwent IA in the PAFC-positive group (40.5% vs. 24.0%, *p* = 0.037); however, no significant difference was observed in the PAFC-negative group (5.3% vs. 6.2%, *p* = 0.71). The postoperative hospital stay was significantly longer in the patients who underwent EA than in those who underwent IA (PAFC-positive: 9.4 ± 5.8 vs. 6.2 ± 4.8 days, *p* < 0.001; PAFC-negative: 5.0 ± 2.9 vs. 3.7 ± 2.7 days, *p* < 0.001). The rate of neoplasia on pathologic examination was significantly higher in the PAFC-positive group than in the PAFC-negative group (7.6% vs. 1.5%, *p* < 0.001); the rate of carcinoma was also higher in the PAFC-positive group (2.4% vs. 0.17%, *p* = 0.02). In patients over the age of 40 years, the rate of neoplasia was 12.2%, and the rate of carcinoma was 3.5% in the PAFC-positive group.

**Table 1:** Patient characteristics and results

	PAFC ( +) (*n* = 170)			PAFC ( −) (*n* = 606)			*p* Value
	EA(*n* = 116)	IA(*n* = 54)	*p* Value	EA(*n* = 509)	IA(*n* = 97)	*p* Value	
Age	47.6 ± 18.6	50.1 ± 17.2	0.42	37.2 ± 16.0	40.2 ± 15.4	0.08	
Sex (M: F)	73:43	26:28	0.07	269:240	42:55	0.08	
BMI	22.5 ± 3.6	22.7 ± 2.9	0.79	22.1 ± 3.6	22.2 ± 4.7	0.9	
*Comorbidities*							
DM (%)	12	7.1	0.63	3	4.8	0.67	
HT (%)	20	21.4	0.92	4.2	19	0.027**	
*Preoperative blood test*							
WBC (*10^3^)	12.7 ± 4.4	8.8 ± 4.4	0.002**	12.5 ± 4.6	7.5 ± 4.2	< 0.001**	
CRP	8.7 ± 8.0	8.7 ± 9.4	0.98	3.0 ± 4.5	2.1 ± 3.3	0.2	
T-Bil	1.2 ± 0.7	0.7 ± 0.3	0.003**	0.9 ± 0.5	0.8 ± 0.3	0.13	
Fecalith on CT (%)	38.2	40	0.86	46.2	24.7	< 0.001**	
Laparoscopy (%)	66.7	94.2	< 0.001**	84.3	94.8	0.01**	
Stump inversion (%)	23.8	8.3	0.02**	15.7	3.4	0.003**	
Drain insertion (%)	50	25	0.005**	4.7	1.1	0.12	
Time (min)	95.4 ± 46.2	81.8 ± 45.5	0.07	66.1 ± 29.9	65.5 ± 35.1	0.87	
Blood (ml)	46.4 ± 103.5	19.8 ± 76.1	0.06	7.2 ± 14	5.5 ± 4.7	0.03**	
Complication (CD ≧ 2) (%)	40.5	24	0.037**	5.3	6.2	0.73	
Postoperative hospital stays (days)	9.4 ± 5.8	6.2 ± 4.8	< 0.001**	5.0 ± 2.9	3.7 ± 2.7	< 0.001**	
Neoplasm (%)	7.6			1.5			< 0.001**
Malignancy + (%)	2.4			0.17			0.002**

Data are shown as mean ± standard deviation, number of patients, or number (percentage)

**p < 0.05

*PAFC (* +*)* positive for periappendiceal fluid collection, *PAFC ( −)* negative for periappendiceal fluid collection, *EA* emergency appendectomy, *IA* interval appendectomy, *M* male, *F* female, *BMI* body mass index, *DM* diabetes mellitus, *HT* hypertension, *WBC* white blood cells, *CRP* C-reactive protein, *T-Bil* total bilirubin, *CT* computed tomography, *Laparoscopy* laparoscopic surgery, *CD* Clavien–Dindo

We also separately analyzed patients who underwent EA and IA. The results of the logistic regression analysis of the factors associated with postoperative complications after EA are presented in Table 2. Only the presence of PAFC was significantly associated with an increased risk of postoperative complications (odds ratio [OR], 7.11; 95% confidence interval [CI], 2.73–18.60; *p* < 0.001). The results of the logistic regression analysis of the factors associated with failure of NOM are presented in Table 3. Only the presence of fecaliths on preoperative CT was significantly associated with an increased risk for the failure of NOM (OR, 24.5; 95% CI, 2.2–273; *p* = 0.009). The presence of PAFC was not a risk factor (OR, 1.48; 95% CI, 0.19–11.7; *p* = 0.71).

**Table 2:** Multivariate analysis of risk factors for postoperative complications after emergency appendectomy

	Odds ratio	95% CI	*p* Value
Age	1.02	0.99–1.05	0.0558
CRP	1.03	0.96–1.1	0.425
WBC	1	1.00–1.00	0.715
PAFC	7.11	2.73–18.6	0.00006*
Time	1.01	1.00–1.03	0.0752
Blood	1.01	0.99–1.02	0.566
Lap	0.31	0.06–1.4	0.133

*p < 0.05

*CI* confidence interval, *CRP* C-reactive protein, *Lap* laparoscopic surgery, *PAFC* periappendiceal fluid collection, *WBC* white blood cells

**Table 3:** Multivariate analysis of risk factors for nonoperative management failure

	OR	95%CI	*p* Value
Age	1.03	0.97–1.09	0.37
Fecalith	24.5	2.20–273.0	0.009*
PAFC	1.48	0.19–11.7	0.71
CRP	0.96	0.83–1.11	0.55
WBC	1	1.00–1.00	0.27

*p < 0.05

*OR* odds ratio, *CI* confidence interval, *PAFC* periappendiceal fluid collection, *CRP* C-reactive protein, *WBC* white blood cells

## Discussion

The treatment strategies for acute appendicitis are controversial [5–8]. EA has not been recommended for complicated appendicitis because it increases the incidence of extended resection and postoperative complications. Some recent reports have recommended NOM even for uncomplicated appendicitis, and repeating NOM in cases of recurrence leads to a reduction of medical expenses [9–11]. However, NOM or IA may be problematic because some cases fail, and repeating NOM may be problematic because some cases of appendicitis are caused by malignant tumors. Failure refers to the requirement for emergency surgery when performing treatment by NOM. These cases should be performed EA at the first decision. However, the cases caused by malignant tumor should be performed appendectomy without complications. In this study, we considered the treatment strategy for acute appendicitis from the following three perspectives: prediction of which patients should avoid emergency surgery, prevention of failure of IA or NOM, and a malignant tumor as the etiology of the appendicitis. We focused on the presence of a PAFC on preoperative CT.

In the PAFC-positive group, the rate of postoperative complications was significantly higher in the patients who underwent EA than in those who underwent IA (40.5% vs. 24.0%, *p* = 0.037). In the PAFC-negative group, however, there was no significant difference between patients who underwent EA and IA (5.3% vs. 6.2%, *p* = 0.71). The multivariate analysis showed that only PAFC positivity was a risk factor for postoperative complications after EA (OR, 7.11; 95% CI, 2.73–18.60; *p* < 0.001). According to a recent report, laparoscopic surgery is associated with few postoperative complications even in patients with complicated appendicitis [12–15]. However, the present study showed that the PAFC-positive group had a higher rate of postoperative complications than the PAFC-negative group regardless of the surgical approach. This difference was likely to have been affected by bias in the surgical procedure depending on the time point of treatment during the study. A prospective study of patients with preoperative PAFC positivity is necessary.

The risk factors for treatment failure have not been clarified [10, 16]. The incidence of NOM failure in patients with complicated appendicitis reportedly ranges from 15.6 to 25.7%, which is higher than that in patients with uncomplicated appendicitis [17–19]. Various methods for diagnosing complicated appendicitis and predicting the risk of NOM failure before surgery, such as scoring systems, have been investigated [20–22]. We examined whether PAFC positivity is a risk factor for NOM failure. Our univariate analysis showed that the failure rate was significantly higher in the PAFC-positive than PAFC-negative group (20.4% vs. 4.1%, *p* = 0.004). However, the multivariate analysis showed no significant difference (OR, 1.48; 95% CI, 0.19–11.7; *p* = 0.71), and the presence of fecaliths on CT was the only risk factor (OR, 24.5; 95% CI, 2.2–273; *p* = 0.009). Of course, various factors are involved in failure; PAFC positivity alone does not substantially increase the risk.

Neoplasms can cause acute appendicitis, and they are difficult to diagnose by preoperative examination alone [11]. The guidelines recommend against routine IA for patients under the age of 40 years [1]. One report indicated that repeated NOM in patients with recurrence leads to a reduction of medical expenses [10, 11]. However, among patients undergoing IA, neoplasia is reportedly found in about 10% of cases by pathological diagnosis [23, 24]. Likewise, in the present study, neoplasia was found in 12.2% and carcinoma in 3.5% of patients aged 40 years or older in the PAFC-positive group. Therefore, the possibility of a tumor should not be forgotten in patients with a PAFC.

This study had two main limitations. First, there was bias in the surgical procedure depending on the time point at which treatment was performed. Laparotomy was common in the first half of the study period, and most procedures were laparoscopic surgeries in the second half. Laparoscopic surgery reportedly reduces complications and may need to be considered separately from laparotomy. Second, the definition of NOM failure has not been determined, and the physician’s judgment has strong influence on the outcome. Although cases of NOM failure were treated as complications of IA in our study, the study design may arguably need to be reconsidered.

## Conclusion

A PAFC on preoperative CT was found to be a risk factor for postoperative complications but not NOM failure. It was also correlated with neoplasia as the etiology of appendicitis. Therefore, PAFC positivity is considered useful for determining the optimal management of acute appendicitis and may be a preoperative indication for IA.

## Data Availability

The datasets used and/or analyzed during the current study are available from the corresponding author on reasonable request.

## Abbreviations

EA: Emergency appendectomy
IA: Interval appendectomy
NOM: Nonoperative management
CT: Computed tomography
PAFC: Periappendiceal fluid collection
